# Circulating follicular T helper cells and cytokine profile in humans following vaccination with the rVSV-ZEBOV Ebola vaccine

**DOI:** 10.1038/srep27944

**Published:** 2016-06-21

**Authors:** Fouzia Farooq, Kevin Beck, Kristopher M. Paolino, Revell Phillips, Norman C. Waters, Jason A. Regules, Elke S. Bergmann-Leitner

**Affiliations:** 1 Malaria Vaccine Branch, Walter Reed Army Institute of Research, Silver Spring, MD 20910, USA; 2 Miltenyi Biotec Inc., San Diego, CA 92121, USA; 3 Clinical Trials Center, Walter Reed Army Institute of Research, Silver Spring, MD 20910, USA; 4 Medical Vaccine Branch, Chemical and Biological Defense Program, Joint Science and Technology Office, Defense Threat Reduction Agency, Fort Belvoir, VA 22060, USA; 5 Department of Clinical Research, United States Army Medical Research Institute of Infectious Diseases, Ft. Detrick, MD 21702, USA.

## Abstract

The most recent Zaire Ebolavirus (ZEBOV) outbreak was the largest and most widespread in recorded history, emphasizing the need for an effective vaccine. Here, we analyzed human cellular immune responses induced by a single dose of the rVSV-ZEBOV vaccine candidate, which showed significant protective efficacy in endemic populations in Guinea. This is the first in-depth characterization of ZEBOV-GP specific, circulating follicular T cells (cTfh). Since antibody titers correlated with protection in preclinical models of ZEBOV infection, Tfh were predicted to correlate with protection. Indeed, the ZEBOV-specific cTfh data correlated with antibody titers in human vaccines and unexpectedly with the Tfh17 subset. The combination of two cutting edge technologies allowed the immuno-profiling of rare cell populations and may help elucidate correlates of protection for a variety of vaccines.

The genus *Ebolavirus* belongs to the family *Filoviridae* (filovirus) and includes a number of highly pathogenic viral species, which can be transmitted to humans from wild animals^1^ and easily from person to person^2^. Ebola virus disease (EVD) is a severe disease in humans, associated with a fatality rate, which has varied from 25 percent to 90 percent over the recorded history of outbreaks^2^,^3^. EVD was first reported in 1976 in two simultaneous outbreaks, in Sudan, and the Democratic Republic of Congo (DRC) where two distinct species of Ebolavirus, Zaire Ebolavirus (ZEBOV) and Sudan Ebolavirus (SEBOV) were associated with the significant outbreaks^4^, however, the most recent outbreak exceeded all previous epidemics in terms of geographic range and number of cases^3^.

Even though this disease is associated with a high fatality rate, prophylaxis and treatment options remain scant and have not been fully evaluated for clinical efficacy. Desirable target product characteristics of a ZEBOV vaccine are high efficacy after a single immunization, rapid-onset of protection, and long-lasting immunity. A vaccine with such properties would have the potential to quickly stop the spread of the disease or even prevent significant outbreaks. Various vaccine platforms have been evaluated for their efficacy in preclinical models including nonhuman primates. One of the lead vaccine platforms is based on a live, replication-competent recombinant vesicular stomatitis virus (rVSV)^5^ in which the gene for the VSV glyco protein (VSV-GP) is replaced by the ZEBOV glycoprotein. This vaccine successfully protected nonhuman primates (NHP) against a challenge with ZEBOV^6^,^7^. Moreover, this vaccine demonstrated post-exposure protection (PEP) in a NHP model, which has led to its investigational use as a countermeasure under contingency protocols for suspected Ebola Zaire exposures as post exposure therapy^8^,^9^,^10^; early clinical trials in the US, Europe, and Africa have demonstrated that a single inoculation of the vaccine candidate is immunogenic^11^,^12^,^13^ and tolerated in the majority of vaccinated subjects although reactogenicity was observed^11^,^12^. A phase III trial of efficacy with ring vaccination design, whereby close contacts of ZEBOV patients were vaccinated immediately or three weeks after diagnosis of the newly identified case, has suggested that this live, attenuated, single-dose vaccine candidate is highly efficacious^14^.

To date, however, reported correlates of vaccine-induced immune protection for EBOV remain varied, with data from preclinical models indicating the involvement of both cellular and humoral mechanisms^7^,^15^,^16^,^17^. The type of immune mechanisms leading to protection may depend on the vaccine platform. Immune mechanisms induced by the rVSV-ZEBOV vaccines have been investigated in mouse models^15^ and in NHP^7^. In the latter, animals were immunized with rVSV-ZEBOV following the depletion of either CD4^+^ or CD8^+^ T cells during immunization or right before challenge. While depletion of CD8^+^ T cells did not affect vaccine efficacy, the loss of CD4^+^ T cells at time of vaccination had a great impact on antibody responses (depressed titers) and the resulting protection. Depletion of CD4^+^ T cells at the time of challenge had no impact indicating that this T cell population has no direct effector function.

The objective of the present analysis was to characterize the circulating follicular helper T cells (cTfh) and cytokine immune profiles induced by the rVSV-ZEBOV vaccine since minimal human data (i.e., only serology) are available on the immune profile induced by this vaccine candidate. The vaccine was originally developed by Public Health Canada, licensed to NewLink Genetics Corp., which initiated clinical testing and GMP manufacturing of the vaccine (designated BPSC1001), and subsequently sublicensed it exclusively to Merck & Co, which is engaged in the late stage development of the vaccine candidate (V920). The current study establishes highly detailed immunoprofiles for a cohort of V920-immunized human subjects (for description of the clinical study, see ref. 13) and the tools and parameters which will allow a comparison with responses in ZEBOV- exposed humans, thus, informing assessments of the immune response that may subsequently be applied to the identification of immune correlates of protection.

## Results

### Cytokine profile of ZEBOV-GP stimulated immune responses in PBMC

For the characterization of the cytokine profile of ZEBOV-GP-specific peripheral blood mononuclear cells (PBMC), culture supernatant from ZEBOV-GP-peptide stimulated cells were analyzed using the Mesoscale cytokine multiplex assay platform (Table 1). The analyzed cytokines are representatives for different functional categories: IL-1β and IL-8 (pro-inflammatory), IFN-γ, IL-12, IL-2, TNF-α (Th1), IL-4, IL-6, IL-13 (Th2), and IL-10 (immuno-modulatory). IFN-γ was the most abundantly secreted cytokine by cells from all three vaccine dose cohorts in response to ZEBOV-GP peptides. The enrolled subjects were divided into three dose groups and received either 3 × 10^6^, 2 × 10^7^, or 1 × 10^8^ plaque-forming units (pfu) of rVSV-ZEBOV as a single intramuscular inoculation, respectively.

**Figure 1:**
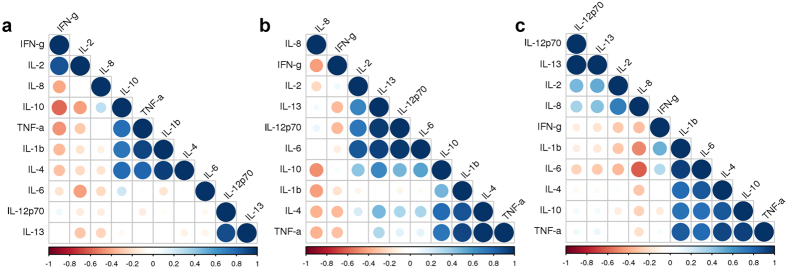
Clustering of net cytokine signatures in response to rVSV-ZEBOV vaccination. Correlogram depicts the relationships between cytokines produced by PBMC from subjects in Cohort 1 (Panel a), Cohort 2 (Panel b) and Cohort 3 (Panel c) in response to stimulation with ZEBOV-GP-peptides. Colors indicate the level of correlation (blue – positive, red – negative, white – no correlation). See Table 1 for actual cytokine data.

Cytokine signatures for each cohort were established using a correlation matrix (Fig. 1) which resulted in the following key observations: (1) in cohort 1, a cluster of correlations results from antigen stimulation that consists of IL-1β, IL-4, IL-10, and TNF-α. There is also a strong correlation between IL-12 and IL-13, but with only two factors, this cannot be considered a true cluster. IFN-γ, while expressed at high levels by stimulated cells, does not have any other correlations. (2) For cohort 2, there are two clusters of cytokines that show correlations: Cluster One consists of IL-2, IL-6, IL-12 and IL-13 while Cluster Two consists of IL-1β, IL-4, IL-10, and TNF-α. IFN-γ has only a negative correlation with IL-8. (3) The clusters in cohort 2: Cluster One consisting of IL-2, IL-8, IL-12, IL-13 and Cluster Two with IL-1β, IL-4, IL-6, IL-10 and TNF-α. IFN-γ still has only negative, albeit, weak correlations, namely with IL-8 and IL-2. We conclude that increasing the vaccine dose changed the cytokine signature of the stimulated PBMCs. While the cluster of IL1β, IL-4, IL-10 and TNF-α is detected in all three cohorts, the basic cluster of IL-2, IL-12, IL-13 only becomes apparent in cohort 2. While IL-6 is part of Cluster One in cohort 2, it shifts into Cluster Two in Cohort 3. There is a strong negative correlation between IL-6 and IL-8. The degree of pro-inflammatory cytokine release increased with the vaccine dose. The dose groups differed in the magnitude of the IL-2 response (p = 0.03, ANOVA, IL-6 (p = 0.016, ANOVA), and TNF-α (p = 0.017, ANOVA). The groups also differed in their IL-8 response albeit not significantly (p = 0.08, ANOVA).

### Immunization with rVSV-ZEBOV-GP induces significant levels of cTFH

**Figure 2:**
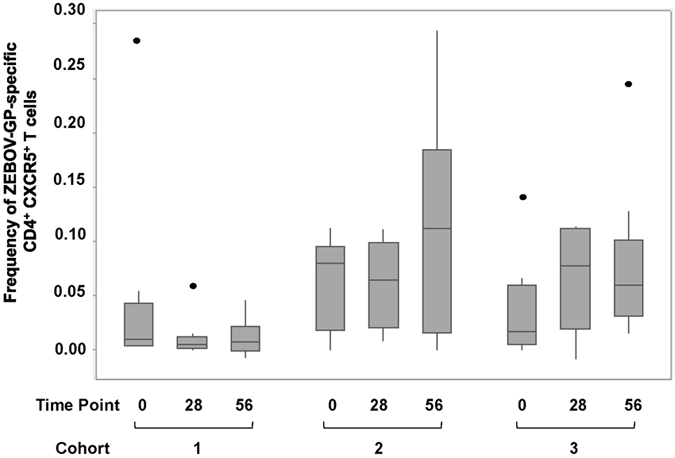
Frequency of ZEBOV-GP-specific cTfh increases after vaccination in peripheral blood. Cohorts 1, 2 and 3 received a single IM inoculation of 3 × 10^6^, 2 × 10^7^ or 1 × 10^8^ pfu, respectively. Lymphocytes from Day 0, Day 28 and Day 56 were stimulated with EBOV peptides and stained for the expression of CD3, CD4, CD154, CXCR5. Box plot represent n = 10 subjects per vaccine cohort and time point. Frequencies reported here are the percentage CD154^+^ (ZEBOV-specific) CD4^+^ CXCR5^+^ cells within viable CD3^+^ population of PBMC. Statistical differences between vaccine cohorts were found on Day 28 (p = 0.004, ANOVA) and Day 56 (p = 0.013, ANOVA).

**Figure 3:**
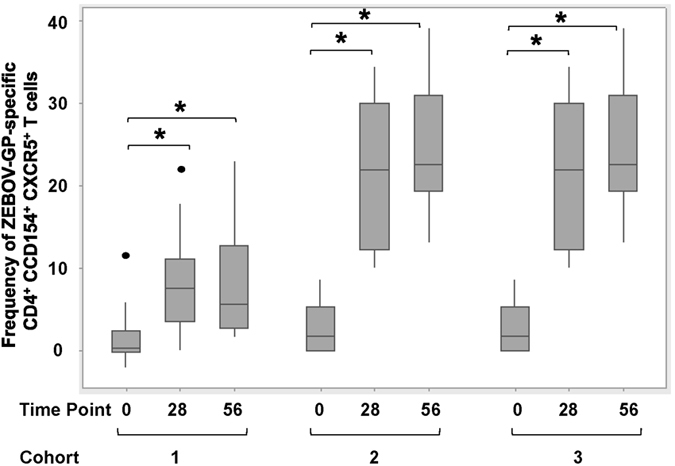
Kinetic of ZEBOV-GP-specific cTfh as a function of vaccine dose and time. Cohorts 1, 2 and 3 received a single IM inoculation of 3 × 10^6^, 2 × 10^7^ or 1 × 10^8^ pfu, respectively. Lymphocytes from Day 0, Day 28 and Day 56 were stimulated with ZEBOV-GP- peptides and enriched based on the expression of activation marker CD154. Box plot represent n = 10 subjects per vaccine cohort and time point. See Supplementary Fig. S1 for gating strategy. The frequency reported is percentage of CXCR5^+^ cells within enriched CD4^+^CD154^+^ T cells. The frequencies were statistically different between the cohorts on Day 28 (p < 0.001, ANOVA) and Day 56 (p = 0.009), but not for Day 0 (p = 0.78). Brackets with asterisks indicate statistical differences between time points. See Supplementary Table S1 for detailed statistical analysis.

The use of CD154 as activation marker and tool for rare cell detection has been described previously^18^. The initial evaluation of ZEBOV-GP-specific CD4^+^ CXCR5^+^ T cells based on the expression of CD154 after stimulation with ZEBOV-GP-peptides revealed that the frequency of cTfh cells was very low (Fig. 2) and that an in-depth analysis of functional subsets within this population required modifying the logistics of the flow cytometric analysis. Responses in placebo control subjects did not exceed the responses in vaccinated subjects measured for Day 0. Thus, PBMCs from Day 0, Day 28 and Day 56 were stimulated with overlapping ZEBOV peptides or control stimulation, and enriched based on the expression of the activation marker CD154. The frequency of CXCR5^+^ CD4^+^ T cells within the enriched population increased in all three vaccine cohorts from baseline levels (Day 0) (Fig. 3). The frequency of the CXCR5^+^ T cells was significantly different in the three cohorts on Day 28 (p < 0.001, ANOVA) and Day 56 (p = 0.009, ANOVA). There was a significant interaction between time point and cohort (p = 0.001, two-way ANOVA) (Supplementary Table S1). No significant changes in the frequencies were observed between Day 28 and Day 56. The high dose cohort (cohort 3) had the highest responses, but whether the higher responses are associated with altered function or persistence of the cTfh cells remains to be determined.

### Vaccine dose and its impact on the differential induction of cTfh subsets

**Figure 4:**
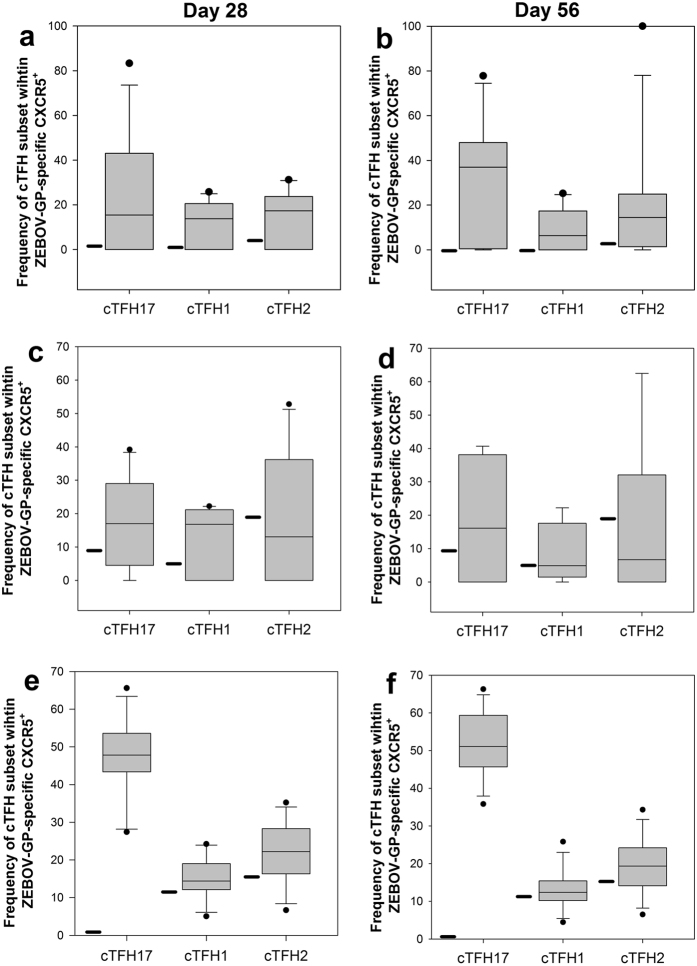
Changes in ZEBOV-GP-specific Tfh subpopulations as a function of vaccine dose and time. PBMC enriched based on the expression of activation marker CD154 were subsequently analyzed by flow cytometry for the expression of CD3, CD4, CXCR5 and the subset specific markers CCR6 and CXCR3. Responses in the various cohorts (cohort 1 (Panel a,b), cohort 2 (Panel c,d), cohort 3 (Panel e,f)) are shown for Day 28 (Panel a,c,e) and Day 56 (Panel b,d,f) in box plots. The bold line next to each box plot represents the median frequency of each cTfh population at Day 0. Data expressed as absolute numbers of CD3^+^CD4^+^CXCR5^+^CCR6^−^CXCR3^−^ (Tfh2), CD3^+^CD4^+^CXCR5^+^CCR6^−^CXCR3^+^(Tfh1), and CD3^+^CD4^+^CXCR5^+^CCR6^+^CXCR3^−^(Tfh17) within CD4^+^CD154^+^ T cells (see Supplementary Fig. S1 for gating strategy). See Supplementary Table S2 for detailed statistical analysis.

To further investigate the differences in the composition of ZEBOV-GP-specific CD4^+^ T cell responses and a potential polarization of the cellular response depending on vaccine dose, we evaluated the expression of chemokine receptors (CXCR3 and CCR6), which are associated with distinct CXCR5 subsets in human peripheral blood^19^,^20^. The analysis revealed a marked predominance of cTfh17 cells, followed by the cTfh2 and cTfh1 cell subset (Fig. 4). Immunization with the rVSV-ZEBOV vaccine resulted in significant increase in the frequency of cTfh17 subset (especially in cohort 3), but not in the other Tfh subsets. When expanding the statistical analysis to include both factors, time point after vaccination and vaccine cohort, there was a significant interaction between these factors for the cTfh17 subpopulation (Two-way ANOVA, p < 0.001), but not for the cTfh1 or the cTfh2 subpopulations (Supplementary Table S2). The kinetics of changes in the subset also demonstrates that there is, at a minimum, maintenance of these Tfh subsets, as the frequencies of the ZEBOV-GP-specific cTfh is maintained at Day 56.

### Correlation between frequencies of cTfh subsets and ZEBOV-GP-specific antibody titers

**Figure 5:**
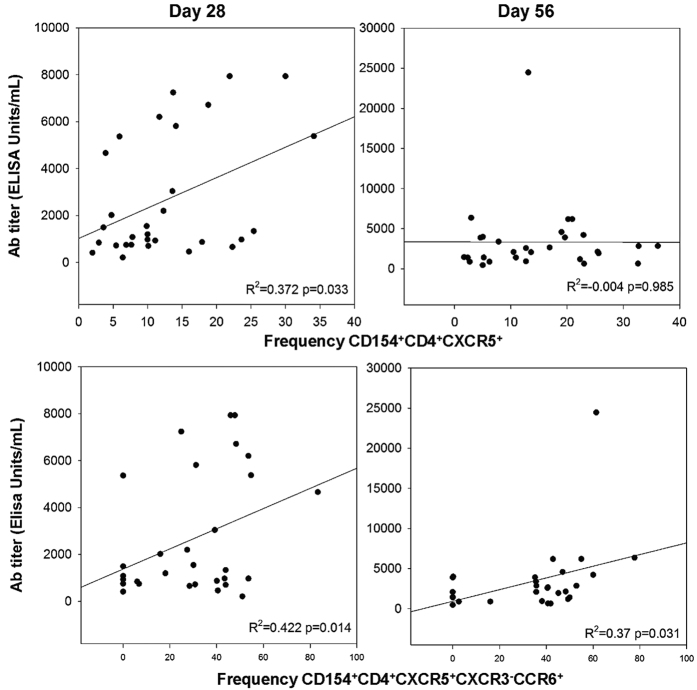
Correlation between frequency of ZEBOV-GP-specific cTfh cells and antibody titers. Scatterplots comparing the ELISA titer (measured as ELISA units/mL) and frequency of CD154^+^CD4^+^CXCR5^+^ (cTfh, top row) or frequency of CD154^+^CD4^+^CXCR5^+^CCCR6^+^CXCR3^−^ (cTfh17, bottom row) at Day 28 (left column) and Day 56 (right column). Pearson correlation coefficient R^2^ and p-values are shown. Data from all cohorts were pooled to achieve a large sample size. No correlations between antibody titer and cTfh1 or cTfh2 were observed (Supplementary Fig. 2).

The comparison of vaccine-induced antibody titers (Supplementary Table S3) with cTfh frequencies revealed a significant correlation between ZEBOV-specific cTfh cells (CD154^+^CD4^+^CXCR5^+^) on Day 28 (R^2^ = 0.372, p = 0.033) (Fig. 5, top row). The correlation is lost at Day 56 and antibody data from later time points are not yet available. When further analyzing the relationship between the subsets of ZEBOV-GP-specific cTfh subsets and antibody titer, an unexpected correlation was found between Tfh17 levels and antibody titers for Day 28 (Fig. 5, bottom row, R^2^ = 0.422, p = 0.014) and Day 56 (R^2^ = 0.37, p = 0.031), but no significance for Tfh1 and Tfh2 and antibody titer (Supplementary Fig. S2).

## Discussion

The present study employs a combination of two exquisitely sensitive technologies, and provides a detailed and first cytokine profile of ZEBOV-glycoprotein (GP)-specific immune responses in humans. Three cohorts of volunteers received a single intramuscular injection of an increasing dose of the rVSV-ZEBOV vaccine. The dose given to the second cohort corresponds to the dose used for the ring trial in Guinea which resulted in a very high level of protective efficacy against infection during the outbreak in 2014. When stimulated *in vitro* with a peptide pool corresponding to sequences from the ZEBOV-GP, the vaccine dose correlated with a broader and more complex cytokine pattern with increasingly complex associations. The responses of cells from the low dose vaccine group (cohort 1) were dominated mainly by IFN-γ secretion. This cytokine increased with the vaccine dose, but did not positively correlate with any other cytokine in any volunteer from either one of the three cohorts (Table 1, Fig. 1). It did, however, negatively correlate with IL-8 in cohort 2 and additionally with IL-2 in cohort 3. The cytokine signatures of the vaccinated subjects in cohorts 2 and 3 revealed two main clusters of correlations: (1) Cluster One with IL-2, IL-12, and IL-13 - depending on the vaccine dose, either IL-6 and IL-10 (cohort 2) or IL-8 (cohort 3) are part of this cluster. Overall, many of these factors can promote T cell and B cell proliferation, independent of each other. (2) Cluster Two contained IL-1-β, IL-4, IL-10 and TNF-α. We hypothesize that an increase in vaccine dose leads towards a skewing in the cytokine profile that promotes humoral immune responses which is supported by the serological data^13^ and Supplementary Table S3. A causative link between these two parameters will need to be established through future functional studies. Additional insight may be gained by characterizing the lineage of the cytokine producing cells. In our study, we employed a highly sensitive bulk detection-method, namely the Mesoscale platform, to analyze culture supernatants from stimulated PBMCs. Such bulk readout methods report cytokine profiles as well as absolute amounts of cytokine at the population level and, therefore, characterize the overall cytokine response of an individual vaccine recipient. The use of multiplexing assays allows a broad characterization of the immune response with a limited amount of cells, but, unlike intracellular cytokine staining, no conclusions can be drawn regarding the frequency and identity of cytokine/chemokine producing cells. However, when analyzing rare cells such as antigen-specific cTfh in limited amounts of sample, their frequency may be below the detection threshold of a flow cytometer (reviewed in ref. 18). Thus, such analyses are typically restricted to few factors and unable to generate broad cytokine profiles. For this study, we sought to establish a readout protocol which could be applied to a variety of vaccine studies in which the amount of patient material is limited, but high-density data sets are required in the search of immune correlates of protection.

Previous studies in preclinical models have suggested that antibodies and CD4^+^ T cells are involved in protection against ZEBOV mediated by the rVSV-ZEBOV vaccine^7^,^15^. Thus, apart from the characterization of the cytokine profile, we sought to gain insights into functional CD4^+^ T cell subsets, namely Tfh cells as these cells are crucial for mediating a T-cell dependent humoral immune response. The present study characterizes functional CD4^+^ T cell subsets based on previously established subsets markers^19^,^20^. Although the precise phenotype of cTfh cells is still subject of discussions, we used a well-established panel^19^ for the characterization of the vaccine-induced cTfh –profile to aid in the immunological assessment of this vaccine candidate. Modification of conventional flow cytometric analysis was necessary as the frequency of antigen-specific cTfh cells was low (Fig. 2), which was anticipated given the immunological naivety of the study subjects to ZEBOV-GP and the immunization with a single dose of the vaccine. To overcome this challenge, we enriched antigen-specific T cells based on CD154 and subsequently performed an in-depth characterization of the antigen-specific CD4^+^ CXCR5^+^ T cell subsets (Fig. 3). We categorized the CD4^+^ T cell subsets based on the expression of CXCR5 (a chemokine receptor which guides migration of leukocytes into the B cell compartments of lymphoid tissues), CXCR3 (expressed on activated T cells, preferentially Th1 cells), and CCR6 (expressed on memory T cells, preferentially Th17 cells)^21^ (Fig. 4). The latter cell population is increasingly being recognized as playing a crucial role in immunity against infectious diseases, and it has been postulated that Th17 cells play a role in vaccine-induced immune memory. The actual distribution of Tfh subpopulations between tissue and peripheral blood has yet to be fully described (reviewed in ref. 22). While scientifically important, ultimately, this type of characterization bears little relevance in the context of any analysis that seeks to describe an immunoprofile to guide vaccine development, as peripheral blood is the most readily accessible source of immune cells for such an effort. Thus, the use of cTfh cells, as opposed to tissue-resident Tfh cells, in the search for correlates of protection, is highly appropriate^22^.

The role of Tfh17 cells in vaccine-induced immunity is an emerging field of research. Until recently, these cells had been associated predominantly with disease progression and pathology, but it is now increasingly understood that they play a crucial role in mediating protection against a variety of pathogens^23^,^24^,^25^ and that they may be induced mainly by live, attenuated vaccines^23^,^26^ and mucosal vaccines^27^. Both Tfh2 and Tfh17 subsets have been shown to be efficient in providing B cell help^20^. Finally, increased frequencies of Tfh2 and Tfh17 cells were detected in individuals who had broadly neutralizing antibodies to HIV^28^ supporting the current hypothesis that Tfh17 in addition to Tfh2 may be important in promoting humoral immune responses. Our data indicate that cellular immune responses are efficiently maintained at Day 56 after a single immunization with various doses of rVSV-ZEBOV (Fig. 4). The focus of future studies should be to delineate the relationship of the present immunological analysis with immunological parameters governed by the interaction of Tfh and B-cells, specifically antibody affinity and Fc functionality, as well as the persistence of memory.

The observation that antibody titers correlated with the frequency of ZEBOV-GP specific cTfh17 cells (Fig. 5) was unexpected since, traditionally, Tfh2 are thought to promote the majority of the humoral response. A few recent studies, however, have demonstrated that IL-17 plays a role in the induction of antibody responses, isotype switching and germinal center formation^29^,^30^. Moreover, there is increasing evidence that Th17 play a crucial role in recall responses induced by vaccines targeting bacterial, fungal and viral pathogens (reviewed in ref. 21). The current study expands this knowledge to human immune responses against ZEBOV, which has not been previously reported. We cannot rule out that other T cell subsets such as Tfh2, especially tissue-resident cells in the germinal centers of lymphatic tissues, also contribute to the antibody response and may, thus, act synergistically with cTfh17. The data presented here are restricted to responses that can be measured in peripheral blood and therefore, establish only a surrogate marker of an efficacious vaccine response against ZEBOV.

Regarding the dose response to the vaccine observed in the present study, there is precedence from other disease models that while immunization with high antigen doses results in higher frequency of antigen-specific cells, the affinity of these cells’ T cell receptor (TCR) is reduced^31^,^32^. A recent study indicates that the antigen affinity greatly impacts the differentiation of T cell responses^33^. We are showing this striking impact of vaccine dose on the Tfh1/Tfh2/Tfh17 ratio for the first time in the context of a human ZEBOV vaccine. The understanding of the relationship between antigen dose, quality of the T cell response and the resulting persistence of Tfh subpopulations is crucial for the design of vaccine regimens and improving vaccine efficacy beyond vaccines just against ZEBOV.

Future studies should aim at expanding the analysis presented in this study using samples from vaccinated subjects in field studies as well as profiling the cytokine and cTfh responses in survivors. Increasing the analysis panel to include assessment of the frequency of ZEBOV-specific memory B cells as well as intensifying the characterization of the antibody response in regards to affinity, isotype profile, and even glycosylation patterns of the immunoglobulin heavy chain (reviewed in ref. 34) will bring us even closer to establishing a definitive correlate of protection against ZEBOV, while the present study represents an important first leap towards this goal.

## Materials and Methods

### Study design and samples

Samples were obtained from subjects participating in the Phase I clinical trial (NCT02269423) conducted at the Clinical Trials Center at WRAIR^13^. The methods were carried out in accordance with the approved guidelines. All experimental protocols were approved by the WRAIR Human Subject Protection Branch (WRAIR#2163) and all study subjects had provided informed consent for future use of the samples. Peripheral blood mononuclear cells (PBMC) were collected on Day 0 and at various time points after vaccination and cryopreserved.

### Pooling of the ZEBOV Kikwit GP Consensus peptides

15-mer lyophilized peptides (overlapping by 11 amino acids) were purchased from Atlantic Peptides (Lewisburg, PA) and reconstituted in DMSO at a concentration of 10 mg/mL. Equal amounts of peptides were pooled thereafter, and divided into 6 equal aliquots (0.45 mg of each peptide); hydrophobic peptides were omitted from the pools.

### Human 10-plex Cytokine Pro-inflammatory Panel

Cryopreserved PBMCs of each study subject from time points Day 0 and Day 28 days post immunization were stimulated with ZEBOV-GP peptide pools (15-mer peptides overlapping by 11 AA) at 0.5 μg/mL final concentration for 48 hours. Mesoscale Discovery’s 10-plex human pro-inflammatory panel kit (IL-1β, IL-8, IL-2, IL-4, IL-6, IL-10, IL-12p70, IL-13, IFN-γ, TNF-α) was used to analyze culture supernatants according to the manufacturer’s protocol. Plates were read using a QuickPlex SQ120.

### Polychromatic Flow Cytometry Staining

Cryopreserved PBMCs from Days 0, 28, and 56 were cultured with a ZEBOV-GP Megapool (pool of all peptide) at 1.0 μg/mL or medium alone (control stimulation). Cells were cultured for 16 hours (37 °C, 5% CO_2_) in complete medium (RPMI-1640 (Life Technologies, Waltham, MA) containing 10% human serum (Gemini Bio-Products, West Sacramento, CA)) at a concentration of 5 × 10^7 ^cells/mL. Anti-human CCR6-APC (REA190) and CD40 (HB14) were added to the culture at a 1:10 and 1:100 dilution, respectively. Following stimulation, cells were washed and stained with anti-human CD154-biotin (5C8) for 15 minutes at 4 °C in FACS solution (0.5% human serum and 0.1% sodium azide in PBS). Cells were further incubated with anti-biotin microbeads (ultrapure, Miltenyi Biotec, San Diego, CA) for 15 minutes at 4 °C. After washing, a pre-titrated and optimized antibody cocktail with fluorochrome-conjugated antibodies against CD3-VioBlue (BW264/56), CD4-PerCPVio700 (M-T466), CD185-PEVio770 (REA103), CD183-VioBrightFITC (REA232), CD154-PE (5C8) and Zombie Aqua Fixable dye (BioLegend, San Diego, CA) was added and incubated for 45 minutes at 4 °C. All monoclonal antibodies for cell culture and analysis were purchased from Miltenyi Biotec (San Diego, CA). After washing and resuspending in PBS, cells were enriched and acquired on a MACSQuant Analyzer 10.

The gating strategy is depicted in Supplementary Fig. 1. Lymphocytes were first gated by scatter, and then based on viability and lineage marker CD3. Enriched antigen-specific cells were gated by co-expression of CD4 and CD154, followed by the expression of CD4 and CXCR5. Within the CD4^+^CXCR5^+^ cells, subsets were further identified - based on CCR6 and CXCR3 expression- as Tfh1, Tfh2, and Tfh17 cells. The quantitative analysis was performed using FlowJo 10 (Treestar, Ashland, OR).

### Statistical analysis

Data from the different cohorts and time points were tested for statistical significance using ANOVA (Minitab software package, Minitab Inc., State College, PA). Correlations between Tfh frequencies and antibody titers at the respective time points (Day 0, Day 28, Day 56) were determined by the Pearson correlation (Minitab). The correlation matrix/correlogram was computed and plotted using R software (STHDA website).

## Additional Information

**How to cite this article**: Farooq, F. *et al*. Circulating follicular T helper cells and cytokine profile in humans following vaccination with the rVSV-ZEBOV Ebola vaccine. *Sci. Rep.* **6**, 27944; doi: 10.1038/srep27944 (2016).

## Supplementary Material

Supplementary Information

## Figures and Tables

**Table 1:** Cytokine/chemokine concentration in culture supernatants from ZEBOV-GP-stimulated PBMC^a^.

Analyte^b^	Cohort 1^c^	Cohort 2^c^	Cohort 3^c^
IFN-γ	26,892 ± 1,157	51,934 ± 459	538,974 ± 41,165^$ #^
IL-10	17 ± 6	26 ± 7	149 ± 93
IL-12p70	234 ± 221	8 ± 4	131 ± 110
IL-13	39 ± 17	49 ± 16	194 ± 112
IL-1β	683 ± 47	516 ± 362	759 ± 206
IL-2	53 ± 17	21 ± 13	415 ± 183^#^
IL-4	46 ± 28	14 ± 5	26 ± 8
IL-6	2,601 ± 180	11,119 ± 3,581^@^	13,726 ± 2,419^#^
IL-8	656 ± 382	1,451 ± 535	3,231 ± 1,281^#^
TNF-α	185 ± 100	333 ± 192	1,255 ± 389^$#^

^a^Data expressed as pg/ml after subtraction of Day 0 responses.

^b^The sensitivity of the various analytes: IFN-γ (0.2 pg/ml), IL-10 (0.03 pg/ml), IL12p70 (0.11 pg/ml), IL-13 (0.24 pg/ml), IL-1β (0.04 pg/ml), IL-2 (0.09 pg/ml), IL-4 (0.02 pg/ml), IL-6 (0.06 pg/ml), IL-8 (0.04 pg/ml), TNF-α (0.04 pg/ml).

^c^Data are the average ± SEM of n = 10 subjects per cohort.

Statistical significances: ^@^p < 0.05 between cohort 1 and 2, ^$^p < 0.05 between cohort 2 and 3, ^#^p <0.05 between cohort 1 and 3.

## References

[b1] Groseth A., Feldmann H. & Strong J. E. The ecology of Ebola virus. Trends microbiol. 15, 408–416, 10.1016/j.tim.2007.08.001 (2007). 17698361

[b2] Leroy E., Baize S. & Gonzalez J. P. [Ebola and Marburg hemorrhagic fever viruses: update on filoviruses]. Med trop (mars). 71, 111–121 (2011). 21695865

[b3] Srivastava P. Update: Ebola Virus Disease Epidemic - West Africa, February 2015. Morb mortal wkly rep. 64, 186 (2015). PMC5779600 25719681

[b4] Muyembe-Tamfum J. J. . Ebola virus outbreaks in Africa: past and present. Onderstepoort j vet. 79, 451, 10.4102/ojvr.v79i2.451 (2012). 23327370

[b5] Lichty B. D., Power A. T., Stojdl D. F. & Bell J. C. Vesicular stomatitis virus: re-inventing the bullet. Trends mol med. 10, 210–216, 10.1016/j.molmed.2004.03.003 (2004). 15121047

[b6] Jones S. M. . Live attenuated recombinant vaccine protects nonhuman primates against Ebola and Marburg viruses. Nat med. 11, 786–790, 10.1038/nm1258 (2005). 15937495

[b7] Marzi A. . Antibodies are necessary for rVSV/ZEBOV-GP-mediated protection against lethal Ebola virus challenge in nonhuman primates. Proc natl acad sci. 110, 1893–1898, 10.1073/pnas.1209591110 (2013). 23319647 PMC3562844

[b8] Feldmann H. . Effective post-exposure treatment of Ebola infection. PLoS pathogens. 3, e2, 10.1371/journal.ppat.0030002 (2007). 17238284 PMC1779298

[b9] Gunther S. . Management of accidental exposure to Ebola virus in the biosafety level 4 laboratory, Hamburg, Germany. J infect dis. 204 Suppl 3, S785–790, 10.1093/infdis/jir298 (2011). 21987751

[b10] Lai L. . Emergency postexposure vaccination with vesicular stomatitis virus-vectored Ebola vaccine after needlestick. Jama. 313, 1249–1255, 10.1001/jama.2015.1995 (2015). 25742465 PMC4874522

[b11] Agnandji S. T. . Phase 1 Trials of rVSV Ebola Vaccine in Africa and Europe - Preliminary Report. New engl j med, 10.1056/NEJMoa1502924 (2015). PMC5490784 25830326

[b12] Huttner A. . The effect of dose on the safety and immunogenicity of the VSV Ebola candidate vaccine: a randomised double-blind, placebo-controlled phase 1/2 trial. Lancet infect dis. 15, 1156–1166, 10.1016/S1473-3099(15)00154-1 (2015). 26248510 PMC6624136

[b13] Regules J. A. . A Recombinant Vesicular Stomatitis Virus Ebola Vaccine - Preliminary Report. New eng j med April 1, published online, 10.1056/NEJMoa1414216 (2015). PMC5408576 25830322

[b14] Henao-Restrepo A. M. . Efficacy and effectiveness of an rVSV-vectored vaccine expressing Ebola surface glycoprotein: interim results from the Guinea ring vaccination cluster-randomised trial. Lancet 386, 857–866, 10.1016/S0140-6736(15)61117-5 (2015). 26248676

[b15] Jones S. M. . Assessment of a vesicular stomatitis virus-based vaccine by use of the mouse model of Ebola virus hemorrhagic fever. J infect dis. 196 Suppl 2, S404–412, 10.1086/520591 (2007). 17940977

[b16] Sullivan N. J. . Accelerated vaccination for Ebola virus haemorrhagic fever in non-human primates. Nature. 424, 681–684, 10.1038/nature01876 (2003). 12904795 PMC7095492

[b17] Sullivan N. J., Martin J. E., Graham B. S. & Nabel G. J. Correlates of protective immunity for Ebola vaccines: implications for regulatory approval by the animal rule. Nat rev microbiol. 7, 393–400, 10.1038/nrmicro2129 (2009). 19369954 PMC7097244

[b18] Bacher P. & Scheffold A. Flow cytometric analysis of rare antigen-specific T cells. Cytometry A. 83A, 692–701 (2013). 23788442 10.1002/cyto.a.22317

[b19] Morita R. . Human blood CXCR5(+)CD4(+) T cells are counterparts of T follicular cells and contain specific subsets that differentially support antibody secretion. Immunity. 34, 108–121, 10.1016/j.immuni.2010.12.012 (2011). 21215658 PMC3046815

[b20] Schmitt N. & Ueno H. Human T follicular helper cells: development and subsets. Adv exp med biol. 785, 87–94, 10.1007/978-1-4614-6217-0_10 (2013). 23456841

[b21] Lin Y., Slight S. R. & Khader S. A. Th17 cytokines and vaccine-induced immunity. Semin immun. 32, 79–90, 10.1007/s00281-009-0191-2 (2010). PMC2855296 20112107

[b22] Ma C. S. & Deenick E. K. Human T follicular helper (Tfh) cells and disease. Immunol cell biol. 92, 64–71, 10.1038/icb.2013.55 (2014). 24145858

[b23] Derbise A., Hanada Y., Khalife M., Carniel E. & Demeure C. E. Complete Protection against Pneumonic and Bubonic Plague after a Single Oral Vaccination. PLoS negl trop dis 9, e0004162, 10.1371/journal.pntd.0004162 (2015). 26473734 PMC4608741

[b24] Gallorini S. . Sublingual immunization with a subunit influenza vaccine elicits comparable systemic immune response as intramuscular immunization, but also induces local IgA and TH17 responses. Vaccine. 32, 2382–2388, 10.1016/j.vaccine.2013.12.043 (2014). 24434044

[b25] Warfel J. M. & Edwards K. M. Pertussis vaccines and the challenge of inducing durable immunity. Curr opin immunol. 35, 48–54, 10.1016/j.coi.2015.05.008 (2015). 26091979

[b26] Warfel J. M., Zimmerman L. I. & Merkel T. J. Comparison of Three Whole-cell Pertussis Vaccines in the Baboon Model of Pertussis. Clin vaccine immunol, 10.1128/cvi.00449-15 (2015). PMC4711092 26561389

[b27] Kumar P., chen K. & Kolls J. K. Th17 cell basedc vaccines in mucosal immunity. Curr opin immunol. 25 (2013). 10.1016/j.coi.2013.03.011 PMC3721632 23669353

[b28] Locci M. . Human circulating PD-1 + CXCR3-CXCR5+ memory Tfh cells are highly functional and correlate with broadly neutralizing HIV antibody responses. Immunity. 39, 758–769, 10.1016/j.immuni.2013.08.031 (2013). 24035365 PMC3996844

[b29] Doreau A. . Interleukin 17 acts in synergy with B cell-activating factor to influence B cell biology and the pathophysiology of systemic lupus erythematosus. Nat immunol. 10, 778–785, 10.1038/ni.1741 (2009). 19483719

[b30] Mitsdoerffer M. . Proinflammatory T helper type 17 cells are effective B-cell helpers. Proc natl acad sci. 107, 14292–14297, 10.1073/pnas.1009234107 (2010). 20660725 PMC2922571

[b31] Bullock T. N., Mullins D. W. & Engelhard V. H. Antigen density presented by dendritic cells *in vivo* differentially affects the number and avidity of primary, memory, and recall CD8+ T cells. J immunol. 170, 1822–1829 (2003). 12574347 10.4049/jimmunol.170.4.1822

[b32] Narayan S., Choyce A., Fernando G. J. & Leggatt G. R. Secondary immunization with high-dose heterologous peptide leads to CD8 T cell populations with reduced functional avidity. Eur j immunol. 37, 406–415 (2007). 17274003 10.1002/eji.200535688

[b33] Keck S. . Antigen affinity and antigen dose exert distinct influences on CD4 T-cell differentiation. Proc natl acad sci. 111, 14852–14857, 10.1073/pnas.1403271111 (2014). 25267612 PMC4205596

[b34] Anthony R. M., Wermeling F. & Ravetch J. V. Novel roles for the IgG Fc glycan. Ann N Y acad sci. 1253, 170–180 (2012). 22288459 10.1111/j.1749-6632.2011.06305.x

