# Supplementary material for: Circulating follicular T helper cells and cytokine profile in humans following vaccination with the rVSV-ZEBOV Ebola vaccine
Source: Sci Rep. 2016 Jun 21;6:27944. doi: 10.1038/srep27944 (PMC4914957; doi:10.1038/srep27944)
Supplement: Supplementary Information [file srep27944-s1.pdf]

Supplementary Materials

Circulating follicular T helper cells and cytokine profile in humans following vaccination with the rVSV-ZEBOV Ebola vaccine

Fouzia Farooq, Kevin Beck, Kristopher M Paolino, Revell Phillips, Norman C Waters, Jason A. Regules, Elke S. Bergmann-Leitner

Supplementary Figure S1

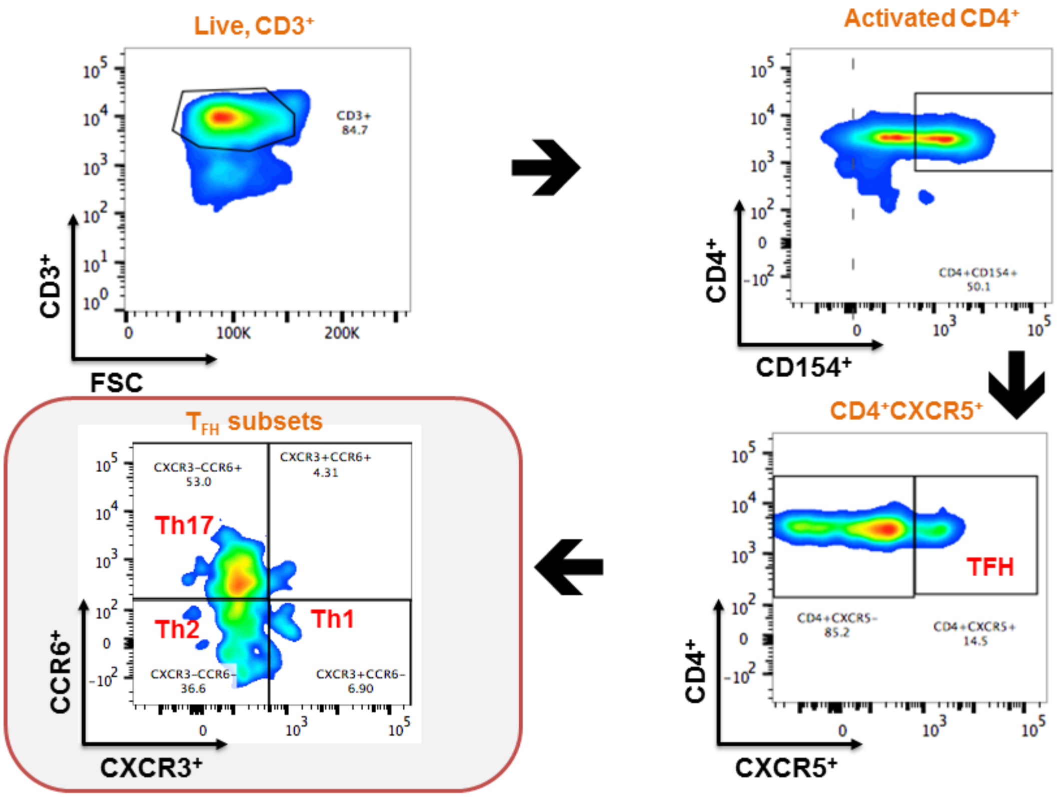

Supplementary Fig. S1. Gating strategy for flow cytometric analysis. After antigen stimulation, cells were gated based on viability (not shown here) and expression of CD3. This population was then further gated based on the expression of activation marker CD154 (cells are considered "antigen-specific") and lineage marker CD4. Antigen-specific $CD4^+CXCR5^+$ cells were then analyzed for the concomitant expression of CXCR3 (Tfh1) and CCR6 (Tfh17).

Supplementary Figure S2

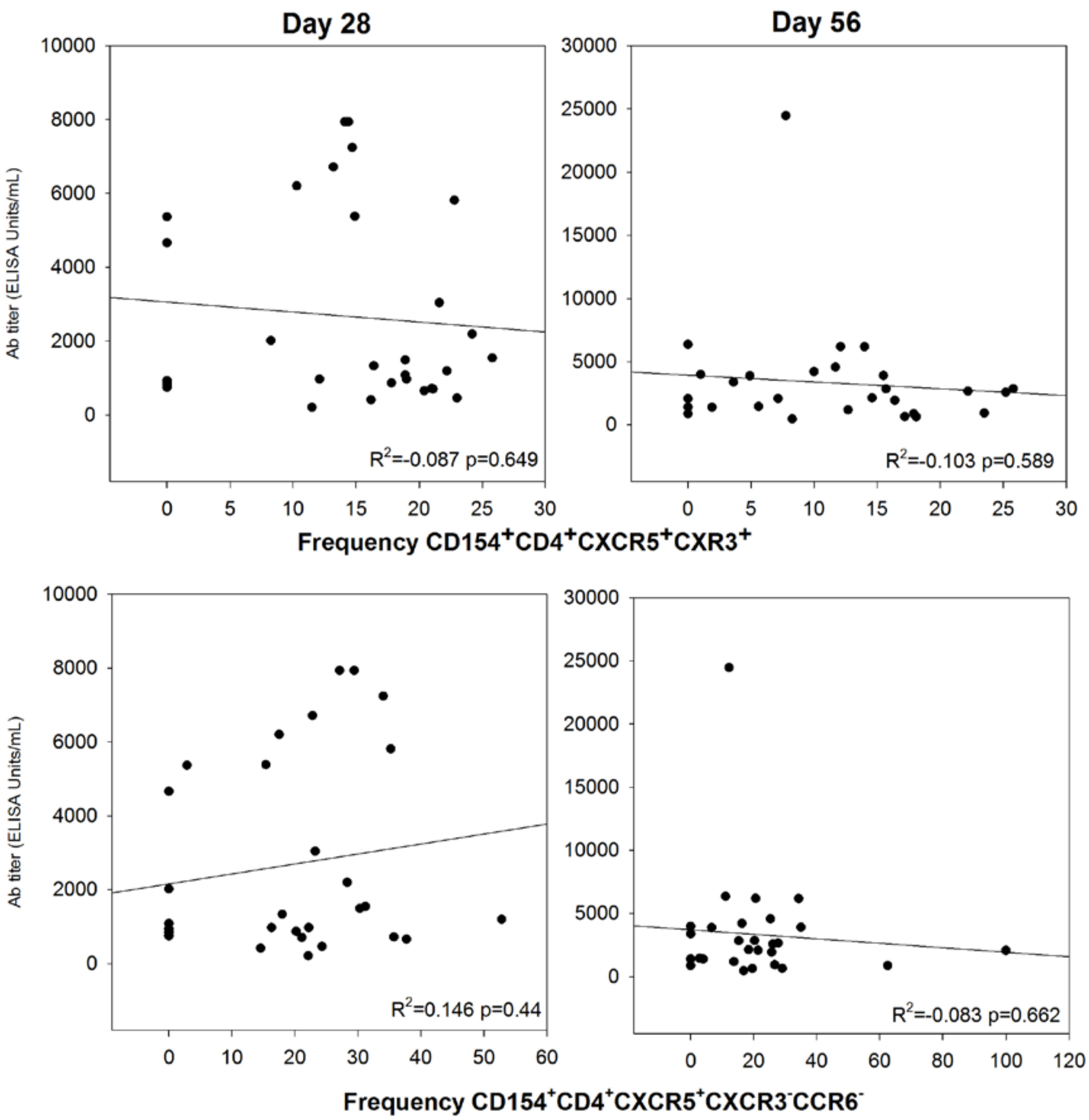

Supplementary Fig. S2. Correlation between frequency of cTFH subsets and antibody titers. Scatterplot comparing ELISA titer (measured as Elisa Units/ml) and frequency of cTfh1 (top row) or cTfh2 (bottom row) at Day 28 (left column) and Day 56 (right column). R^2 and p-values are shown. Data from all cohorts were pooled to achieve a large sample size.

Supplementary Table S1: Frequency of ZEBOV-GP-specific cTfh as function of time and vaccine dose [a]

Time Point	Cohort 1[b]	Cohort 2[b]	Cohort 3[b]
Day 0 vs Day 28	0.078	<0.001	<0.001
Day 0 vs Day 56	0.072	<0.001	<0.001
Day 28 vs Day 56	0.135	NS	NS

Cohort	Day 0[b]	Day 28[b]	Day 56[b]
Cohort 1 vs Cohort 2	NS	<0.001	<0.001
Cohort 1 vs Cohort 3	NS	<0.001	<0.001
Cohort 2 vs Cohort 3	NS	NS	NS

[a] Two-way ANOVA was performed using a general linear model consistent of the frequency as dependent variable and cohort and time point as source of variation. There was a significant interaction between cohort and time point.

[b] Statistical significance (p-value)

Supplementary Table S2: Frequency of ZEBOV-GP-specific cTfh17 as function of time and vaccine dose[a]

Time Point	Cohort 1[b]	Cohort 2[b]	Cohort 3[b]
Day 0 vs Day 28	NS	NS	<0.001
Day 0 vs Day 56	0.02	NS	<0.001
Day 28 vs Day 56	NS	NS	NS

Cohort	Day 0[b]	Day 28[b]	Day 56[b]
Cohort 1 vs Cohort 2	NS	NS	NS
Cohort 1 vs Cohort 3	NS	0.02	0.02
Cohort 2 vs Cohort 3	NS	0.004	0.001

[a] Two-way ANOVA was performed using a general linear model consistent of the frequency as dependent variable and cohort and time point as source of variation. There was a significant interaction between cohort and time point (p=0.001).

[b] Statistical significance (p-value)

Supplementary Table S3: Serological response to Zaire-Kikwit Glycoprotein[a]

Cohort	Study Group[b]	Day 28	Day 56
1	Vaccine, $3x10^6$ PFU	856<br>(95-1616)	1437<br>(377-2498)
2	Vaccine, $2x10^7$ PFU	2104<br>(592-3616)	2527<br>(1736-3318)
3	Vaccine, $1x10^8$ PFU	3280<br>(1662-4898)	3649<br>(2409-4889)

[a] Data expressed as geometric mean (95% CI) of ELISA units/mL as described previously[13]

[b] Detailed clinical study plan and analysis described previously[13]
